# Use of the Canadian CT head rule for patients on anticoagulant/anti-platelet therapy presenting with mild traumatic brain injury: prospective observational study

**DOI:** 10.3389/fneur.2024.1327871

**Published:** 2024-04-18

**Authors:** Laura Uccella, Cristiana Riboni, Francesco Polinelli, Carola Biondi, Graziano Uccheddu, Roberta Petrino, Pietro Majno-Hurst

**Affiliations:** ^1^Emergency Department—EOC—Ospedale Regionale di Lugano, Lugano, Switzerland; ^2^Surgery Department—EOC—Ospedale Regionale di Lugano, Lugano, Switzerland

**Keywords:** anticoagulants, anti-platelet, brain concussion, brain injury, Canadian CT head rule, GCS 15, mild traumatic brain injury

## Abstract

**Background and importance:**

Mild traumatic brain injury (mTBI) is a frequent presentation in Emergency Department (ED). There are standardised guidelines, the Canadian CT Head Rule (CCHR), for CT scan in mTBI that rule out patients on either anticoagulant or anti-platelet therapy. All patients with these therapies undergo a CT scan irrespectively of other consideration.

**Objective:**

To determine whether standard guidelines could be applied to patients on anticoagulants or anti-platelet drugs.

**Design, settings, and participants:**

1,015 patients with mTBI and Glasgow Coma Score (GCS) of 15 were prospectively recruited, 509 either on anticoagulant or anti-platelet therapy and 506 on neither. All patients on neither therapy underwent CT scan following guidelines. All patients with mTBI on either therapy underwent CT scan irrespective of the guidelines.

**Outcome measure and analysis:**

Primary endpoint was the incidence of post-traumatic intracranial bleeding in patients either on anticoagulants or anti-platelet drugs and in patients who were not on these therapies. Bayesian statistical analysis with calculation of Confidence Intervals (CI) was then performed.

**Main results:**

Sixty scans were positive for bleeding: 59 patients fulfilled the criteria and 1 did not. Amongst patients with haemorrhage, 24 were on either therapy and only one did not meet the guidelines but in this patient the CT scan was performed before 2 h from the mTBI. Patients on either therapy did not have higher bleeding rates than patients on neither. There were higher bleeding rates in patients on anti-platelet therapy who met the guidelines vs. patients who did not. These rates overlapped with patients on neither therapy, meeting CCHR.

**Conclusion:**

The CCHR might be used for mTBI patients on either therapy. Anticoagulants and anti-platelet drugs should not be considered a risk factor for patients with mTBI and a GCS of 15. Multicentric studies are needed to confirm this result.

## Introduction

Traumatic brain injury (TBI), defined as brain function impairment due to external forces (1, 2) resulting in loss of consciousness, amnesia or disorientation (3) is one of the commonest occurrences at the Emergency Department (ED) worldwide (4).

TBI is classified in severe (GCS ≤ 8), moderate (GCS from 9 to 13) and mild (GCS ≥ 14) (5).

Whilst there is evidence about the need of a head CT scan for patients with a moderate or severe TBI, there is still discussion on when a patient with mild traumatic brain injury (mTBI) should undergo CT. Several guidelines exist, the most important of which is the Canadian CT Head Rule (CCHR) (6, 7), to assist in deciding when further diagnostic investigation is required for mTBI (1). The CCHR for patients with mTBI is 99%–100% sensitive in detecting patients needing a neurosurgical intervention, but is lacking in specificity (39%–51%) (8). The incidence of intracranial bleeding in patients with GCS 15 who meet the criteria for the rule is about 5%–8% (9). The CCHR was derived excluding people on anticoagulant or anti-platelet medication assuming all those with any TBI symptoms (loss of consciousness, amnesia, etc.) would require CT imaging. There was no explicit comment on the need for CT imaging in the context of head injury without clear evidence of TBI in people with anticoagulant and anti-platelet medication. A recent systematic review could not identify robust empirical data to inform recommendations in this population (10, 11).

Historically anticoagulant and anti-platelet drugs have been considered a risk factor in traumatic brain injury. The American College of Emergency Physicians (2008) states that the management of patients on anticoagulants is unclear and gives no specific recommendations (8). The latest update of the NICE Head Injury Guidelines (11) recommend a head CT for any medium risk patient (GCS 15 within 2 h of injury with history of loss of consciousness or amnesia) taking any anticoagulant or anti-platelet regime – excluding aspirin monotherapy. Where no loss of consciousness or amnesia has occurred shared decision making rather than a mandatory CT brain scan is recommended. The consequence is that every patient on either anticoagulants or anti-platelet drugs with a mTBI undergoes a CT scan.

The number of patients on anticoagulant or anti-platelet therapy is increasing. This is mainly due to the increase in the ageing population. The incidence of hospital presentation for mild TBI will also be a significant issue in an ageing population.

The total anticoagulant prescription nearly doubled from 2014 to 2019 in the UK (15.0 million doses vs. 33.0 million doses) (8). Around 43 million adults in the US (19.0%) took aspirin at least three times per week for more than 3 months in 2010. This was an increase of 57% in aspirin use compared with 2005 (12).

The resulting increasing number of patients on these therapies presenting with mTBI makes it necessary for the clinician to weigh the risk of haemorrhage and the risk of irradiating the brain which can lead to radiation-related damage, as well as the costs of performing unnecessary examinations (13–15).

Intracranial bleeding represents the most feared complication in patients under antithrombotic agents, since it is associated with high morbidity and mortality. There is, however, limited evidence of the role of these drugs on mortality after mTBI (4, 16, 17). Literature suggests that anti-platelet and anticoagulant therapy increase the risk for intracranial haematoma and its progression after mTBI (16, 17), but these evidence is based on patients using vitamin K antagonists (VKAs) or anti-platelet drugs. In the last few years, direct oral anticoagulants (DOACs) diffusion has led to the knowledge that these drugs could be safer than VKAs also in the setting of mTBI (18).

The present study aims at determining whether there is a difference in intracerebral haemorrhage rates in patients with GCS 15 taking either antithrombotic or anticoagulant therapy in the mild TBI patients vs. those not on either therapy as an independent risk factor. If this is the case, CCHR could be applied to these patients, reducing the exposure to unneeded radiation. This will help define the management of mTBI in patients under anticoagulant or anti-platelet treatments.

## Materials and methods

### Study design and study period

This is a mono-centre prospective cohort observational study, involving patients’ charts data collection from adults presenting with mTBI (that is head trauma resulting in loss of consciousness, amnesia or disorientation) to the Emergency Department from the 29th April 2021 to the 31st June 2022.

The study received the approval of the local Ethics Committee and the participants signed an informed consent. The study was conducted in accordance with the declaration of Helsinki.

### Sample size calculation

A sample size calculation was undertaken. Null hypothesis was that there are no differences in bleeding between patients with mild brain injury and GCS 15 on anticoagulant/anti-platelet therapy vs. patients with mild brain injury and GCS 15 not on anticoagulant/anti-platelet therapy. To test this null hypothesis, we had to assume a difference between patients who were and were not on either therapy. We assumed 5%, as this is the incidence of intracranial bleeding in patients neither on anticoagulants nor on anti-platelet drugs meeting CCHR (5). The sample size was calculated to have 80% power, 95% confidence level and 2% margin of error. This sample size was 457 subjects for each group. Therefore, we planned to recruit at least 914 patients.

### Participants description

We included all the adult patients presenting during the study period with mTBI and GCS 15, both on anticoagulants or anti-platelet drugs and neither meeting criteria for mild traumatic brain injury. Exclusion criteria were: medical cause of head trauma (e.g., syncope, epilepsy…), GCS 2 h after trauma of 14 or less, presence of seizures after injury, pregnancy, delayed presentation (>24 h), not having taken regular anti-platelet or anticoagulant therapy, absence of written consent.

The regularity of taking anticoagulant or anti-platelet therapy was ascertained by interviewing patients, family members and family physicians.

Patients not on therapy were included in the study only when meeting CCHR criteria for performing a head CT scan.

One month after the access to ED, enrolled patients received a follow-up phone call to find out their condition.

### Outcome measures

Primary endpoint was the incidence of intracranial bleeding in patients either on anticoagulants or anti-platelet drugs and in patients who were not on these therapies.

Secondary endpoints were:

- the need for intervention after post traumatic head bleeding in the two groups;
- the reliability of CT Head Rule in patients on either anticoagulants or anti-platelet drugs;
- to assess whether anticoagulants and/or anti-platelet drugs are a risk factor for patients presenting with mTBI and GCS of 15; and
- to compare mortality and morbidity after mTBI in the two groups with a follow-up period of 1 month.

### Data validation

Two months were selected randomly for data validation. An independent research collaborator was identified to determine the number of patients who should be included in the study within the data collection period and check if any were missed or added unrightfully. This collaborator was not involved in the initial data collection. No differences were detected.

### Data analysis

Statistical analysis was performed using the open source packages “Pandas,” “NumPy,” “SciPy,” “Seaborn,” and “PyMC” for Mac Os X versions 1.4.1, 1.21.2, 1.7.3, 0.11.2, and 3.11.14, respectively. Statistical significance was considered achieved based on highly credible intervals of parameter estimates and *p* < 0.05. Confidence intervals (CI) were calculated at 95%.

Since we needed to compare proportions of haemorrhages in different sub-populations, we performed both a two-tail t-test computation based on classic proportion comparison using Fischer exact test, and a Bayesian estimate of the parameter distribution of a Bernoulli stochastic variable to model bleeding occurrences using a non-informative uniform prior distribution over the interval 0–1. The estimate was obtained by using the Metropolis-Hastings algorithm in a Markov-chain Monte Carlo (MCMC) model, with a burn-in of 5,000 iterations and runs lasting 40,000 iterations. Traces were inspected to verify convergence diagnostics (Geweke plots and Raftery-Lewis analysis). The posterior parameter distribution was then plotted in order to have a graphical overview and confidence intervals were estimated. Furthermore, by sampling the posterior distributions, we were able to estimate both probability that the parameters describing one population would be different from each other as well as the estimate confidence intervals for Relative Risk (19).

## Results

Between April 2021 and June 2022, 1,015 patients were enrolled, 509 on either anticoagulants or anti-platelet drugs and 506 on neither.

Personal data, causes of injury, met criteria for CCHR, presence or absence of haemorrhage at CT, need for surgical intervention, reason for anticoagulant/anti-platelet therapy, are summarised in Tables 1, 2.

INR values of 37 out of 52 patients anticoagulated with VKA were recorded. The mean value was 1.78 (range 1.2–6.6), 3 patients had a subtherapeutic value (≤1.5), 18 patients a therapeutic value (1.5 < INR < 2.5) and 16 patients an overtherapeutic value (≥2.5).

Of the 1,015 CT scans performed 60 resulted positive for haemorrhage (5.9%).

We considered a CT scan positive when there was any trace of blood, even the smallest (even one single petechia). Of these positive patients, 24 were patients on either anticoagulants or anti-platelets and 36 on neither. Amongst the 60 patients who resulted positive for haemorrhage at CT scan, only one seemed not to meet the criteria for the CCHR. This was a 74 year old patient on aspirin (in primary prevention) who accidentally fell from her height and hit the back of the head. She arrived at the hospital by ambulance 30 min after the accident and GCS assessment was performed at the arrival (GCS 15). CT scan was performed at 50 min from the fall and was positive for subdural haematoma. Thirty minutes after she became confused and did not recognise her son (GCS 12 E3 V3 M6). She underwent neurosurgical intervention as a consequence of the positive CT scan for subdural haematoma.

The remaining 59 positive patients met CCHR (Table 3) criteria for head CT, had minimal bleeding, remained stable at the next CT check-up and did not require surgery. Anti-platelet and anticoagulant therapy was discontinued (with the exception of one patient on warfarin therapy for a mechanical mitral valve, who was anticoagulated with unfractionated heparin and closely monitored, with no progression of minimal subarachnoid haemorrhage detected on CT).

**Table 3:** Canadian CT head rule criteria—from the work of Stiell et al. (6).

High risk	Medium risk
GCS^*^ < 15 at 2 h after injury	Amnesia before impact ≥ 30 min
Suspected open or depressed skull fracture	Dangerous mechanism (pedestrian, occupant ejected, fall from elevation)
Any sign of basal skull fracture	
Vomiting ≥ 2	
Age ≥ 65 years	

CT Head is only required for mild head injury patients with any one of these findings—Rule is not applicable for patients on anticoagulants or with bleeding disorders. ^*^GCS, Glasgow Coma Score.

**Table 1:** Description of sample.

Characteristics	*n* = 1,015
Female	50.5%
Age yr	100%
18–65	34.9%
66–80	25.4%
>80	39.6%
Cause of injury	100%
Fall whilst walking	76.1%
Fall >3 m	6.1%
Road accident	9.6%
Aggression	3.4%
Other	4.6%
Anticoagulated patients (nr)	245
VKA	52
of which met CT Head Rule criteria	40
DOACs	189
of which met CT Head Rule criteria	135
Heparin	4
of which met CT Head Rule criteria	3
Reasons for anticoagulation	100%
Atrial fibrillation/flutter	71.0%
Pulmonary embolism	2.8%
Deep vein thrombosis	5.3%
Other—not known	20.8%
Patients on anti-platelet drugs (nr)	264
Aspirin	227
of which met CT Head Rule criteria	174
2nd generation anti-platelet drugs	37
of which met CT Head Rule criteria	35
Reasons for anti-platelet drugs	100%
Ischemic heart disease	68.9%
Primary prevention	22.7%
Other	8.3%
Haemorrhage at CT scan (nr)	60
Of which anticoagulated patients	14
Who met CT Head Rule criteria	14
Who did not meet CT Head Rule criteria	0
Of which anti-platelets patients	10
Who met CT Head Rule criteria	9
Who did not meet CT Head Rule criteria	1
Neurosurgical intervention (nr)	1
If haemorrhage and no surgery, stability to subsequent CT (nr)	59
Deaths (nr)	0

Personal data, causes of injury, met/non-met criteria for CCHR, presence/absence of haemorrhage at CT, need for surgical intervention, reason for therapy.

**Table 2:** Description of sample by confounding factors.

	Anticoagulants		Anti-platelets		Neither therapy
	DOACS	VKA	ASA	2nd generation anti-platelet	(Neither anticoagulants nor anti-platelets)
Median age (yr)	83	83	82	84	59
>64 yr. (nr-%)	180-95%	48-92%	206-90%	37-100%	231-45%
High energy trauma (nr-%)	12-6%	6-11%	12-5%	3-8%	18-3.5%
>30′ amnesia (nr-%)	3-1.5%	4-7.5%	11-5%	2-5.5%	28-5.5%
Low fall (nr-%)	162-85%	42-80%	187-82%	37-100%	204-40%

Patients on anticoagulant or antiplatelet therapy appear older.

No reversal agents were administered to any patient with a positive scan due to the scarcity of bleeding.

There was no difference in terms of bleeding in the two groups, on anticoagulant/anti-platelet therapy and patients on neither. The two CI greatly overlapped.

At 1 month follow-up we could reach all patients but 20 with a telephone call: 18 did not answer and for two patients phone number was missing. No patient had died or suffered complications following trauma. One patient on DOACs with negative CT scan had suspended rivaroxaban and suffered from ischemic stroke 3 days later. Two patients on neither therapy returned to the ED after 4 and 7 days, reporting headache and neck pain. Investigations revealed no complications and they were discharged home. One last patient on neither drug reported paraesthesias in all four limbs after trauma: an MRI of the spine ruled out major complications.

Of the 509 patients on anticoagulants/anti-platelets, 387 met inclusion criteria for CCHR.

The comparison of patients undergoing either therapy who did and did not fulfil the criteria of the CCHR was statistically significant, as patients who fulfilled the criteria had a higher probability of haemorrhage (*p* = 0.023, CI 4.0%–9.1% for fulfilled and 1.0%–4.9% for unfulfilled criteria).

Amongst participants who met CCHR criteria, the comparison between patients who did take anticoagulants or anti-platelet drugs and the patients on neither was not statistically significant. The two groups overlapped.

**Figure 1:**
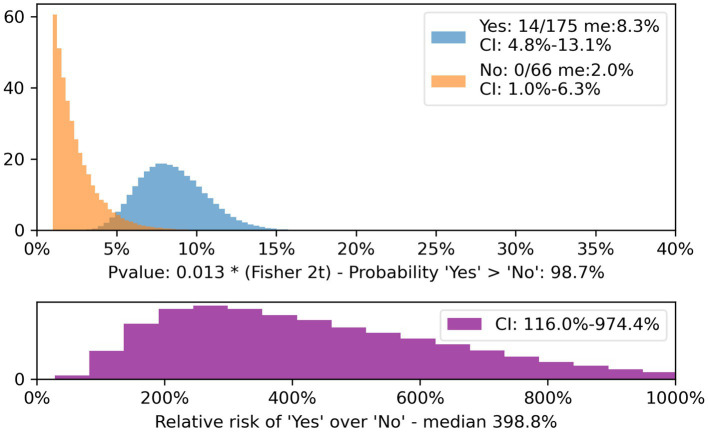
Patients on anti-platelets meeting and non-meeting CT Head Rule, CI and relative risk. The population of patients on anti-platelet drugs who did not meet the CT Head Rule criteria (yellow) is compared with those who did (blue).

When separating the categories of anti-platelets and anticoagulants, the difference in bleeding rate of those who did and did not meet the criteria for the Rule was statistically significant for anti-platelets (*p* = 0.013 CI 4.8–13.1% for met criteria and 1.0–6.3% for non-meet criteria; Figure 1).

The population that met the CCHR criteria had a significantly higher rate of intracranial bleeding and not statistically significant for anticoagulants (CI 2.2%–8.0% for met criteria and 1.1%–6.2% for non-met criteria) with a trend towards more bleeding for patients on anticoagulants who met the criteria.

Comparing anticoagulated patients who met the Rule with patients on anti-platelets who met the Rule, the difference was not statistically significant, with a tendency towards more haemorrhages in patients on anticoagulant drugs.

The comparison between patients anticoagulated with vitamin K antagonists (VKA) and anticoagulated with direct oral anticoagulants (DOACs) was also not statistically significant: the two categories overlapped.

### Data availability

Data from this study are available on https://datadryad.org/stash.

## Discussion

This study shows that anticoagulant or anti-platelet therapies are not an independent risk factor for brain haemorrhage in GCS 15 patients and that the CCHR might be used for patients with mTBI undergoing these treatments.

In the literature mTBI has so far been discussed under the assumption that all patients on anticoagulant or anti-platelet therapy were at high risk, even if they had a GCS of 15 (8, 11, 20). Many retrospective studies evaluated the incidence of bleeding in anticoagulated patients, whilst this paper is giving an answer in a prospective study (4, 16–18).

The results reported are of high importance and likely to impact clinical practice in the ED.

The question is not about whether anticoagulants and anti-platelets are actually a risk factor for haemorrhage in brain injury, even when mild. Indeed, it seems quite clear (although the studies are mostly retrospective) that taking these therapies does carry with it a certain increased risk of developing intracranial bleeding after trauma (21–23).

With this regard, several authors emphasise that anti-platelets vs. anticoagulants, and amongst the latter, VKAs vs. DOACs, are at higher risk (4, 13, 22, 24–26).

The question is whether patients with GCS of 15 within 2 h after trauma should be considered in the same way as other patients. The present work shows that this might be possible. If 2 h after trauma they have maintained an intact neurological state, they should be considered low-risk patients because it is unlikely to be severe damage inside the brain (27). In fact, with regard to patients on either therapy, in this study the probability of bleeding even when meeting the criteria remained comparable to that of patients on neither therapy meeting the same criteria. As for patients on anticoagulants, it appears that those with GCS 15 do not have a higher bleeding rate even when selected using the Rule criteria.

Considering possible confounding factors (median age, percentage of patients > 64 years old, percentage of high-energy incidents, amnesia > 30 min, percentage of low energy traumas), patients on anticoagulant or anti-platelet therapy appear older.

The two groups are also comparable in terms of low energy trauma (with a slight tendency to higher energy trauma for the anticoagulants/anti-platelet group) and amnesia.

We did not proceed with further calculations because, given their older age and similar energy of traumas, they are theoretically at an increased risk of ICH. Since the comparison between anticoagulants/anti-platelets and patients on neither therapy yielded similar results, this reinforces our findings.

In the entire study population, one patient needed surgery for evacuation of a subdural haematoma and she was discharged without neurological sequelae, resuming her normal activity in 1 month. Retrospective analysis of the emergency department management of this patient revealed that head GCS score was registered early. In fact, it had been performed upon the patient’s arrival in the emergency department. At 2 h after the trauma (the time pointed out by CCHR to assess GCS), the patient was no longer GCS 15 and thus theoretically should have been excluded from the study.

With regard to the other 58 patients whose CT was positive for haemorrhage, CT control was stable in all cases. All patients were discharged without neurological sequelae and resumed their normal activity.

Our results are giving an answer to the question whether CCHR is reliable also for patients on anticoagulant and anti-platelet treatment.

More than the immediate symptoms after a mTBI (amnesia, disorientation, transient loss of consciousness), a normal neurological state after 2 h is important, regardless of the treatment the patient is taking.

It is possible to speculate, that the vast majority of CTs performed on GCS 15 patients, even when they meet the Rule’s criteria, are unnecessary, with the exception of patients on anti-platelet therapy.

### Limitations

Our study has some limitations:

- We were not able to measure antiXa activity in the vast majority of patients on DOACs, so we did not actually know their coagulation status, even though the patients we enrolled were regularly taking their therapy. Similarly, we did not assess the platelet function of patients on anti-platelet therapy. Adherence to therapy seemed to us a good surrogate as DOACs level measures are not routinely requested for these therapies. However, this needs further investigation in the context of traumatic intracranial haemorrhage.
- This is a single-centre study that needs confirmation on several sites.
- We were not able to contact 20 patients on follow-up. However, negative outcomes in these 20 individuals are really unlikely (control CT in hospital was stable) and could hardly have changed the outcome of the study.
- This study analysed a group of anticoagulant or anti-platelet medication users combined: bigger studies are needed that analyse anticoagulants alone and anti-platelet drugs alone.

## Conclusion

The CCHR could possibly be used for mTBI patients on anticoagulant or anti-platelet therapy, although the number of diagnostic tests requested with the help of this Rule is probably still too high. Multicenter studies are needed to reinforce this opinion.

Anticoagulants and anti-platelet drugs should not be considered *per se* a risk factor for patients with mTBI and a GCS of 15; the need for CT scan should be weighed against the guidelines used for patients on neither therapy.

## Data availability statement

Raw data from this study is in the supplementary material.

## Ethics statement

The studies involving humans were approved by Comitato etico del Canton Ticino. The studies were conducted in accordance with the local legislation and institutional requirements. The participants provided their written informed consent to participate in this study.

## Author contributions

LU: Conceptualization, Data curation, Formal analysis, Investigation, Methodology, Project administration, Supervision, Writing – original draft, Writing – review & editing. CR: Conceptualization, Investigation, Methodology, Writing – original draft. FP: Conceptualization, Validation, Writing – original draft. CB: Data curation, Methodology, Writing – review & editing. GU: Data curation, Formal analysis, Writing – review & editing. RP: Data curation, Formal analysis, Writing – review & editing. PM-H: Conceptualization, Project administration, Supervision, Writing – review & editing.
